# Cochlear implantation (CI) for prelingual deafness: the relevance of studies of brain organization and the role of first language acquisition in considering outcome success

**DOI:** 10.3389/fnhum.2014.00834

**Published:** 2014-10-17

**Authors:** Ruth Campbell, Mairéad MacSweeney, Bencie Woll

**Affiliations:** ^1^Deafness Cognition and Language Research Centre, University College LondonLondon, UK; ^2^Institute of Cognitive Neuroscience, University College LondonLondon, UK

**Keywords:** cochlear implantation outcome, prelingual deafness, late learning of sign language, critical period for language development, auditory critical period, cortical organization of language in deafness, functional decoupling in auditory cortex

## Abstract

Cochlear implantation (CI) for profound congenital hearing impairment, while often successful in restoring hearing to the deaf child, does not always result in effective speech processing. Exposure to non-auditory signals during the pre-implantation period is widely held to be responsible for such failures. Here, we question the inference that such exposure irreparably distorts the function of auditory cortex, negatively impacting the efficacy of CI. Animal studies suggest that in congenital early deafness there is a disconnection between (disordered) activation in primary auditory cortex (A1) and activation in secondary auditory cortex (A2). In humans, one factor contributing to this functional decoupling is assumed to be abnormal activation of A1 by visual projections—including exposure to sign language. In this paper we show that that this abnormal activation of A1 does not routinely occur, while A2 functions effectively supramodally and multimodally to deliver spoken language irrespective of hearing status. What, then, is responsible for poor outcomes for some individuals with CI and for apparent abnormalities in cortical organization in these people? Since infancy is a critical period for the acquisition of language, deaf children born to hearing parents are at risk of developing inefficient neural structures to support skilled language processing. A sign language, acquired by a deaf child as a first language in a signing environment, is cortically organized like a heard spoken language in terms of specialization of the dominant perisylvian system. However, very few deaf children are exposed to sign language in early infancy. Moreover, no studies to date have examined sign language proficiency in relation to cortical organization in individuals with CI. Given the paucity of such relevant findings, we suggest that the best guarantee of good language outcome after CI is the establishment of a secure first language pre-implant—however that may be achieved, and whatever the success of auditory restoration.

## Background: sensitive periods for cortical processing of (speech) sounds

The advent of pediatric Cochlear implantation (CI) is a great achievement in alleviating the impact of profound prelingual hearing loss (Archbold and Meyer, 2012). In children who have received implants in infancy (under 18 months) extending into early childhood (<3 years) the ability to communicate effectively through spoken language, with good speech processing abilities, is often far in advance of that predicted for a deaf child who does not have a CI. As age and duration of deafness increase, the positive effects of CI become less predictable, although CI can still be extremely effective in some cases (Geers et al., 2011; Markman et al., 2011). In this paper, we focus on CI in relation to prelingual deafness. For these children who have never heard speech, CI provides a new entry point into language. By contrast, children who had useable hearing at birth, and who lose their hearing in late infancy/early childhood (postlingually deafened), may have residual representations of speech acquired from before they lost their hearing. In this case, early hearing experience may have provided an effective “scaffold” to enable the new acoustic input from the CI to map to extant speech representations^1^, and the impact of CI may then be related to the efficiency of such processes.

In CI, the 20,000 hair cells of the human cochlea are replaced with up to 22 electrodes which transduce environmental sound waves arriving at the ear into the electrical code required by the brain. Recreating the auditory signal is technically challenging, as is the ability of higher auditory centers to process the degraded signal (Middlebrooks et al., 2005). Extensive rehabilitation is required in order to achieve optimal auditory function. Auditory cortex is a densely interconnected system, in which reciprocal connections between primary auditory cortex within Heschl’s gyrus (A1) and association areas including secondary auditory cortex (A2) along the superior lateral surface of the temporal lobe, are established through patterned firing. On the basis of animal studies it has been proposed that “functional decoupling” of auditory cortex^2^ underpins poor CI outcome (Kral et al., 2005).

Speech processing in humans with normal hearing is associated with multiply interconnected superior temporal together with frontal opercular regions. Together, these form the perisylvian circuits. In typical development, the increasing interconnections between neural assemblies within these regions also include feedback to subcortical auditory structures, which play an important role in determining the subsequent cortical neuronal architecture (Kral and Sharma, 2012). Animal studies show that both dystrophic and neuroplastic changes occur in congenital deafness which may lead to functional decoupling of components within this system. Dystrophic changes include abnormal synaptic development, as an inevitable consequence of auditory deprivation. This may be partially reversible by early implantation (Kral and Sharma, 2012). Neuroplastic changes correspond to cortical reorganization following a period of sensory deprivation, so that areas previously dedicated to processing in one modality then acquire new functions (Neville and Bavelier, 2002; Sharma and Dorman, 2006; Sharma et al., 2009; Olulade et al., 2014). In general terms, these factors are thought to have long-term effects which are hard to reverse. It is generally accepted that there are several sensitive periods for different phases of sensory development and for different language domains (Neville and Bavelier, 2002; Newport et al., 2002). Nevertheless, the first 3 years of life constitute an extremely important phase for first language acquisition. Early CI is therefore recommended in order to resume a quasi-normal pattern of development of auditory cortex before the closure of a sensitive developmental period (and see Lyness et al., 2013, for further discussion).

Current practice in relation to speech training for deaf children preparing for CI and post-implant rehabilitation varies. All emphasize *auditory* rehabilitation therapy (learning to listen) as critical to good language outcome. Alongside this, the role played by other, visual, language input sources in relation to outcome is strongly de-emphasized. Thus, the current authoritative “An Educator’s Guide, Chapter 3”^3^ states that

*“**Auditory therapy** for the child combined with a home environment that encourages the family to take advantage of every possible opportunity to use spoken language is critical to the implanted child’s progress…* (In classroom communication).. *an auditory-oral placement in which the child…*(is) *using spoken language exclusively has been shown to have a significant effect on the auditory development of a child with a cochlear implant…”* (p. 13).

Some regimes and rationales are, if anything, more extreme, using an argument from neuroscience to support practice. The claim is that exposure to nonauditory communicative signals should be minimized because of their assumed deleterious effects on the dynamic development of auditory cortical circuits. Thus, in some “auditory-verbal” training regimes the speaking model focusses on training the child’s acoustic skills by reducing (hiding) the visibility of oral actions, and parents may be advised not to develop their child’s sign language skills prior to implantation (Chan et al., 2000; Rhoades and Chisholm, 2001; Yoshida et al., 2008; Giezen, 2011 for review). This type of clinical practice can be seen to follow a neurological hypothesis which suggests that the deaf child should not watch speech or a sign language, since this may disrupt auditory cortical development during the sensitive period “..*studies of deaf children have demonstrated that* (when) *CI is less effective*.. (it) *appears to be related at least in part to communication through sign language, because of cortical reorganization of the auditory cortex”* (Charroó-Ruíz et al., 2013, p. 20).

Is such advice warranted? Here we outline neurophysiological and psychological reasons to suggest that the costs of depriving the deaf prelingual infant of non-acoustic communicative signals prior to implantation are considerable and are not warranted by the neurophysiological evidence. The evidence cited in favor of prioritizing acoustic stimulation at the cost of other (visual) communicative systems in early childhood can admit explanations which may reflect other factors responsible for poor outcomes with CI. We highlight that one of these may be inadequate acquisition of a signed language as a first language rather than exposure to such signals.

## Does exposure to visual stimulation result in functional decoupling of auditory cortex in the deaf infant brain?

Pioneering neuroimaging studies suggested that prelingual hearing loss was associated with relative isolation of A1 from higher auditory centers. Following a study by Okazawa et al. (1996), which used PET in post-implant adults to assess activation to heard speech in auditory cortical regions, Naito et al. (1997) contrasted post-implant cortical organization in prelingually and postlingually deafened adults. Whereas in postlingually deaf patients, heard speech activated both primary (A1) and secondary (A2) auditory cortex, in prelingually deaf (*n* = 5) heard speech only activated A1. A similar finding was reported by Nishimura et al. (1999) in a single case study, and by Hirano et al. (2000) in a group study of pre- and postlingually deafened adults, implanted in mid-childhood or later.

This apparent decoupling of A1 from higher auditory centers was assumed to reflect abnormal synaptic development in A1; development which could be further adversely affected by exposure to aberrant projections from nonauditory (i.e., visual) stimuli within the isolated A1. This assumption was supported by human neuroimaging studies indicating A1 may be activated by non-linguistic visual stimuli in deaf but not in hearing adults (Finney et al., 2001, 2003; Fine et al., 2005). Thus, exposure to any non-auditory signal by usurping projection sites in primary auditory cortex was thought to lead to functional decoupling of the cortical network for speech processing. In the hearing brain, these regions support acoustic speech processing. The benefit of CI would therefore be greatest when brains have been shielded from non-auditory stimulation. By contrast, least benefit would be gained when children with CI were exposed to non-auditory stimuli (and see Lyness et al., 2013, for further discussion).

## Neural activation associated with CI outcomes

The neuroimaging technique of F-FDG-PET can be used to measure metabolic activity (a proxy for synaptic density) in the resting brain. This can be prognostic of CI success (Catalán-Ahumada et al., 1993). An influential study (Lee et al., 2001), which measured resting brain metabolic state in 15 prelingually deaf children prior to CI, associated hypometabolism (low resting state brain metabolism) in A1 and A2 with good CI outcome, as assessed by auditory speech perception proficiency.

*“..In our prelingually deaf patients who performed poorly with their cochlear implants, the auditory cortex was probably incapable of perceiving auditory signals from the implants. The realm of afferent neural networks of other sensory systems, such as the visual or somatosensory system, might have increased. Alternatively, higher cognitive functions such as the interpretation of sign language or lip-reading could have occupied the relatively under-utilized areas of the auditory cortex.... If cross-modal plasticity restores metabolism in the auditory cortex before implantation, prelingually deaf patients will show no improvement in hearing function, even after successful implantation and concentrated rehabilitation. The resting cortical metabolism of untreated prelingually deaf patients represents a usurping by cross-modal plasticity..” (p. 150)*.

However, later studies suggested a very different relationship between resting state metabolism and post-implant success in prelingually deaf individuals. Lee et al. (2007a) and Giraud and Lee (2007) report post-implant speech perception as a function of pre-implant metabolic state in 22 prelingually deaf children^4^. They found that the best predictor of auditory speech perception 3 years post implant was pre-implant metabolic activity: hyper metabolism (increased metabolic activity) in left dorso-lateral prefrontal cortex and angular gyrus was related to better auditory speech perception outcomes. Additionally, pre-implant metabolism in auditory association regions was highly correlated with duration of deafness (longer duration of deafness, greater hypermetabolism). Initial reports of hypometabolism in auditory (A1 and A2) regions associated with good post-implant outcomes (Lee et al., 2001; Oh et al., 2003) were therefore not upheld for prelingually deaf children^5^. Since duration of deafness is associated with poorer CI outcome (in addition to hypermetabolism in A1 and A2), the recommendation for early CI for all deaf children is still valid—although the relevance of metabolic state of specific brain circuitry is unclear. The variable findings concerning the relationship between resting metabolic state and CI outcome cannot provide a firm basis for the idea that “visual takeover” of auditory brain regions may be responsible for poor outcomes in prelingual CI patients.

Electrophysiological evidence has also been used to support the claim that activation of auditory cortex by non-auditory stimuli during a developmentally sensitive period is responsible for functional decoupling of auditory cortical circuits, and thus leads to poor CI outcomes in prelingual deafness (Gordon et al., 2011; Kral and Sharma, 2012). In ERP studies, the latency of the P1 cortical auditory evoked potential can be considered a marker for the maturity of auditory pathways (Sharma et al., 2002). P1 latency has been proposed to correspond to the time-course for synaptic propagation through central and peripheral auditory pathways (Eggermont, 1988; Ponton et al., 2000). This component has an established developmental trajectory in hearing children, which enables inferences to be drawn concerning the maturity of auditory pathways in children who have undergone CI. P1 was studied in 104 congenitally deaf children, who varied in duration of deafness from 1.3 to 17.5 years (Sharma et al., 2002). After periods of auditory deprivation of less 3.5 years, and 6 months of CI stimulation, the evoked potential was comparable to that of hearing children. By contrast, in those who had had 7 years of auditory deprivation, P1 latency never returned to normal, even after extensive periods of electrical stimulation. From this, the authors suggested a critical period of 0 to 3.5 years for effective CI, after which outcomes tend to be poorer (Sharma et al., 2002; Kral and Sharma, 2012). Whilst the emphasis of that work is on the failure of typical synaptic development in early deafness, the authors claimed that visual takeover of auditory cortex is one of the degenerative changes which reduce the chances of successful CI.

## Re-examining the “visual takeover” hypothesis: a direct test

The “visual takeover” hypothesis proposes that the colonization of A1 by visual projections may contribute to functional decoupling of elements within the auditory processing network—and thence to poor CI outcomes for prelingually deafened individuals exposed to such material. So, for example, with regard to preparation for and rehabilitation with CI, the aim is “..*to limit activity which restricts auditory function”* (Gordon et al., 2011, p. 217).

Since the consequences of this proposal for clinical practice and rehabilitation are profound and far-reaching, the validity of the assumption on which it is based requires reappraisal. In this, as in many other findings for which claims concerning rehabilitation are made, inconsistencies in the literature, and inferences from them, need to be drawn with care. For example, while activation in response to moving dot stimuli has been reported in *primary* auditory cortex (A1) for deaf but not for hearing people (Fine et al., 2005; Finney et al., 2001, 2003), this finding is inconsistent amongst the now numerous neuroimaging studies of deaf adults processing visual input. The majority of subsequent studies have not reported robust activation in A1 in deaf participants for visual input (e.g., MacSweeney et al., 2002; Leonard et al., 2012)^6^. It is also at odds with consensus in the animal literature that primary auditory cortex is only marginally implicated (if at all) in visual processing in deaf cats (Kral et al., 2006; Lomber et al., 2010; Meredith et al., 2011; Barone et al., 2013). The finding of activation for visual stimuli in A1 of deaf participants is also inconsistent, with clear negative findings by Giraud and Lee (2007), who found no correlation between pre-implant metabolic status in A1 and post-implant auditory processing proficiency, and who concluded that *“primary auditory regions do not appear to re-organize in a cross-modal manner…”* (p. 386). It is not clear, then, whether and to what extent regions considered as A1 in the hearing brain are susceptible to non-auditory signals in the (prelingually) deaf brain.

Since A1 is activated by CI in both pre- and post-lingually deaf individuals (see above), the argument concerning the relationship between nonauditory cortical stimulation and poor CI outcome shifts decisively to one focusing on functional decoupling between primary and secondary auditory cortex, and/or “malfunction” within A2, rather than a failure consequent on abnormal projections to, and activation within, A1 itself.

## Activation by visual signals in secondary auditory cortex: sensitivity to deafness

Since direct evidence for visual activation of A1 in deaf brains appears to be weak, the next question is whether secondary (and association) auditory cortex, A2, is activated by such stimuli and whether, if this can be shown, this constitutes a negative prognosis for CI with respect to learning the spoken language. In deaf people who are sign language users, activation of A2 has been robustly demonstrated in response to sign language (Neville et al., 1998; Petitto et al., 2000; MacSweeney et al., 2002, 2004, 2008b; Sakai et al., 2005; Corina et al., 2007; Capek et al., 2008b; Emmorey et al., 2011; Cardin et al., 2013), biological motion including non-linguistic gesture (MacSweeney et al., 2004; Corina et al., 2007; Campbell et al., 2011); and moving dot stimuli (Finney et al., 2001, 2003; Sadato et al., 2004; Fine et al., 2005; Vachon et al., 2013). Petitto et al. (2000) hypothesized that these areas are responsive to phonologically structured input. Emmorey et al. (2011) confirmed that phonetically well-structured, but meaningless American Sign Language (ASL) signs activated this region in deaf native signers, but not in non-signers. However, activation of posterior superior temporal regions is not confined to linguistic analysis. Rather, this region appears to be tuned to respond to the processing of a wide range of intentional actions (that is, actions which have potential communicative import) performed by plausible (social) agents in whatever modality they occur, and however “incomplete” the stimulus presentation (Hein and Knight, 2008; Nummenmaa and Calder, 2009). Thus, for instance, MacSweeney et al. (2004) found that British Sign Language (BSL) and Tic-Tac, an idiosyncratic manual-brachial gestural code used by UK race-course bookmakers, both elicited activation in A2, despite the lack of linguistic structure in Tic-Tac. Deafness may amplify this sensitivity, whatever the stimulus material.

## Speechreading: a special case?

Speech, as well as being heard, can also be seen as movements of the face and mouth (speechreading or lipreading). In hearing people, seen facial actions dubbed to heard speech can lead to illusory speech perceptions (McGurk effects), and can modulate the perception of heard speech, for instance in improving the perception of speech in noise (Campbell, 2008). In people born deaf, despite its inadequacy in capturing the full range of articulated speech, speechreading can offer a route into spoken language. Does seen speech use the same cortical circuits as those for heard speech? If so, these may provide the grounds for auditory speech processing post-CI.

The first neuroimaging study of silent speechreading in hearing adults claimed that seen silent speech activated lateral parts of the superior temporal plane, including A1 (Calvert et al., 1997). This claim was disputed (Bernstein et al., 2002), but appears to have been supported by studies using 3T fMRI in subject-by-subject analysis (Pekkola et al., 2005). Activation within A1, however, is not of identical extent to that seen for heard speech. In any given individual, speechreading typically activates only some small regions within Heschl’s gyrus, towards the lateral edge (e.g., Calvert et al., 1997, 2000; Pekkola et al., 2005). A different approach, contrasting audiovisual and (purely) auditory speech activation also suggests that adding clear vision to audition can enhance activation in A1 (see e.g., Calvert et al., 2000; Reale et al., 2007).

Speechreading thus appears to offer a counterexample to the proposal that (in hearing people) visual stimuli never activate A1. One explanation is that auditory imagery evoked by seen speech generates the activation in primary auditory cortex (Meyer, 2011). In this case, A1 may be less susceptible to activation by seen speech in deaf brains. However, in both prelingually deaf (Capek et al., 2008b, 2010) and recently-deafened adults (Lee et al., 2007b), seen speech generated extensive activation throughout auditory cortex. In prelingually deaf adults, activation in these regions was somewhat more extensive than for hearing respondents, extending into parts of Heschl’s gyrus (Capek et al., 2010). Our conclusion, then, is that regions said to constitute A1 on the basis of studies of auditory speech alone may actually include regions which are sensitive to bi-modal speech (seen as well as heard speech).

In contrast to relatively restricted (in comparison to heard speech) activation by speechreading in A1, secondary auditory cortex—A2—extending from the mid superior temporal gyrus to the temporo-parietal boundary and inferior parietal regions, is reliably activated by silent speechreading in hearing and in deaf individuals (Calvert et al., 1997, 2000; Ludman et al., 2000; Campbell et al., 2001; MacSweeney et al., 2002; Calvert and Campbell, 2003; Hall et al., 2005; Sakai et al., 2005; Skipper et al., 2005; Lee et al., 2007b; Reale et al., 2007; Capek et al., 2008a,b, 2010); the more attentive (Pekkola et al., 2006) and skilled (Capek et al., 2010) the speechreader, the more activation is observed in this region. Moreover, many studies of people with normal hearing attest to the critical role of this supramodal region in modulating auditory perception for congruent audiovisual speech and for incongruent audiovisual tokens—including tokens that generate illusory perceptions. Such McGurk effects, where seeing “ga” and hearing “ba” can lead to the perception of “da” (McGurk and MacDonald, 1976), all implicate posterior superior temporal regions within A2 as a dynamic “hub” for such audiovisual integration (e.g., Calvert et al., 2000; Jones and Callan, 2003; Sekiyama et al., 2003; Miller and D’Esposito, 2005; Bernstein et al., 2008; Beauchamp et al., 2010; Stevenson et al., 2011; McGettigan et al., 2012; Szycik et al., 2012).

It is hard to avoid the conclusion that posterior superior temporal regions, comprising secondary auditory cortex in hearing people, are not only critical for the integration of heard and seen speech, but are highly and dynamically responsive to speech, irrespective of modality. Lee et al. (2007b) consider this region to show “latent multimodal connectivity” for speech. That is, A2 can be readily and immediately activated by one modality when the other is absent.

## Visible and audiovisual speech in CI

The question then arises: does A2 continue to support the effective processing of speech when it is delivered to A1 by CI? Two possibilities present themselves: either visual speech interferes with the new auditory signal to A1, reflecting reduced cross-modal plasticity in A2, and supporting the functional decoupling hypothesis, or, by contrast, A2 supports the integration of visual speech with projections from the newly activated A1 to deliver good (auditory) CI outcomes.

To test these different possibilities, correlations between speechreading and post-CI auditory speech processing should be informative, as would indications of the cortical sites of audio-visual integration in prelingually deaf CI-implantees compared with people with normal hearing. Hirano et al. (2000), reported A2 activation when speechreading but not when listening, in two prelingually deaf patients with CI, and their speech recognition (auditory vowel identification) was not subsequently improved by CI. In contrast, a single participant who was not a good lip-reader showed activation in A2 during listening, but not speechreading, and improved auditory identification post CI. The authors argued that pre-CI speechreading delivered poorest outcomes in relation to CI, supporting the functional decoupling hypothesis. However, there are reasons to be skeptical. A sample size of three, with few details concerning the language background of the implantees and with many potential sources of variability, as well as an unusual test of auditory speech processing, suggest that these findings may not generalize. Moreover, when auditory and visual information were presented together, participants reported as good lipreaders out-performed those reported as poor lipreaders.

While this is the only reported study of prelingually deaf patients which has explored pre-implant speechreading and post-implant auditory speech processing, there is plenty of indirect evidence to suggest that, far from interfering with auditory speech processing, silent speechreading experience can *enhance* auditory speech processing post-CI. As might be expected, postlingually deafened people tend to be better speechreaders than their hearing peers and more reliant on visual speech pre-implant than people with normal hearing (Tyler et al., 1997). They are also, crucially, more effective integrators of vision and audition following CI (Rouger et al., 2007, 2008) and following a period of adjustment to the degraded signal from the implant, cortical organization for speech perception is similar in CI implantees to that of normally hearing controls (Rouger et al., 2012)^7^.

In behavioral studies, pre-implant speechreading in prelingually deaf children was a good predictor of post-implant auditory speech processing abilities (Bergeson et al., 2005). In prelingually deaf infants with very early (10–24 months) CI, sensitivity to audiovisual speech congruence, while initially distinctive, soon came to resemble the pattern seen in children with normal hearing (Bergeson et al., 2010).

Thus, while one case study suggests a negative relationship between (pre-implant) speechreading skill and post-implant auditory success, most studies point to a positive correlation between speechreading and good CI outcomes, and hence do not support functional decoupling. In general, adolescent and adult CI patients continue to be relatively more dependent on visual inputs when processing audiovisual material than people with normal hearing^8^. As far as the cortical correlates of speech processing are concerned, larger scale studies, along the lines of (Hirano et al., 2000) study, are required to establish whether prelingually deaf people show functional decoupling in A1 and A2 in relation to vision (speechreading) and audition (hearing) and in audiovisual speech processing. Here, it will be important to establish the extent to which A1 activation by silent speech, pre-implant, specifically impacts on CI outcome. The functional decoupling hypothesis predicts a negative impact. Behavioral studies to date (albeit with post-lingually deaf adults) suggest no such outcome (i.e., Rouger et al., 2008). The relatively greater reliance on vision in deaf people, while apparent in relation to a range of tasks including speechreading, does not appear to impact negatively on the functional specialization of putative auditory regions and their interaction with visual processes. If anything, rather than vision and language interfering with audition in CI, correlations between patterns of neural activations in visual cortex and increasingly successful subsequent performance with CI suggest the opposite. Experience with the implant resulted in greater and more stimulus specific activation in visual areas (V1, V2, MT and inferior temporo-occipital regions) and auditory association areas (Giraud et al., 2001; Giraud and Truy, 2002; Lazard et al., 2011). In contrast, over time, activation in primary auditory cortex increased, but did not become more stimulus specific (Giraud et al., 2001). It is highly likely that the skilled deaf speechreader post-implant will not use the same cortical circuitry as a person with normal hearing: her experiences with speech have been very different. But this does not mean that her speech processing will be less efficient. Larger scale studies are needed to explore the extent to which A1 activation post-implant correlates with speech processing capabilities in the CI population. For example, it is well established that speechreading is especially useful in discriminating visible place of articulation (Summerfield, 1979) and this is a phonetic feature which is difficult to capture with the limited frequency sensitivities delivered by CI (as suggested by Giraud et al., 2001).

## Consequences and correlates of impaired first language learning on cortical organization and CI

If the evidence for functional decoupling is weak, how are we to explain poor outcomes of CI in some prelingually deaf patients? Here, we suggest that poor CI outcomes may reflect late and incomplete acquisition of a first language, which in turn is reflected in anomalous cortical organization. In prelingually deaf people, a first language may be a sign language, such as ASL or BSL, or a spoken language acquired through speechreading^9^. On this argument, the neural correlates of late first language acquisition may provide clues about causes and clinical management of speech acquisition with CI.

Prior to CI, one of the most important sources of cognitive and linguistic variability in deaf children was the hearing status of their parents. Deaf children from multigenerational deaf families acquire a native sign language and share a rich, linguistically structured communicative environment with their earliest caregivers. However, most deaf children (at least 95%) are born to hearing parents and could not benefit from a natural, language-rich environment in their early years. The development of language and communication in the deaf child of hearing parents can be severely compromised as a result (Mayberry, 2007, 2010; Marschark and Spencer, 2009). The critical period hypothesis (Lenneberg, 1967) suggests that if a child fails to learn language before the end of childhood s/he will never reach the normal level of mastery, with full command of syntax, phonology and verbal working memory. Do such late language learners, who constitute the vast majority of prelingually deaf people, show atypical structural and functional circuitry for language processing as adults?

Mayberry et al. (2002) explored mastery of sign language in late learning deaf adults born to hearing parents. These people, who had failed to develop spoken language skills in an oral environment, were exposed to sign language in mid- to-late-childhood or even later, often in a Deaf school. Although they appeared to use sign language fluently, language tests showed that effective syntactic processing remained rudimentary, and morphological and phonological skills were relatively poor, leading to reduced working memory spans and more generally impaired language mastery (Mayberry, 2010). The comparison group for these late sign learners were native signers who were deaf children of deaf parents. While comprising only around 5% of deaf children, they readily develop (a signed) language at similar ages and stages to the acquisition of a spoken language in their hearing, speaking peers (Meadow-Orlans et al., 2004; Mayberry, 2010; Woolfe et al., 2010). Cortical circuitry for processing the linguistic aspects of sign in such native signers essentially recapitulates that for hearing people who process their first spoken language “by ear” (e.g., Neville et al., 1998; Petitto et al., 2000; MacSweeney et al., 2002, 2004; see MacSweeney et al., 2008a). To date, no studies have explored the neural and cognitive correlates of delayed language acquisition in children who are late sign learners, and those few studies with adult late-sign learners point to a very different pattern of cortical activation in late learners than in native learners of the language, with reduced activation in the perisylvian circuitry for language tasks in the late sign learners (see e.g., Ferjan Ramirez et al., 2013; Leonard et al., 2013).

MacSweeney et al. (2008b) reported that making decisions about sign-language phonology activated left inferior frontal regions in native signers to a greater extent than in late sign learners. In testing syntactic and phonological perception skill for sign language, Mayberry et al. (2011) found activation varied as a function of when the 22 deaf participants learned sign as their first language.

“..*Consistent with previous neurolinguistic research, the sign language processing of individuals born deaf whose age onset of language acquisition began in early life showed neural activation in the brain’s classic language regions. By contrast, the sign language processing of those individuals born deaf whose age-onset of language acquisition began well beyond infancy, and who acquired little functional spoken language in the interim, showed neural activation patterns that deviated from the classic one in a systematic fashion..*” There were “*..negative effects in anterior brain regions responsible for higher level linguistic processing and positive ones in posterior brain regions responsible for lower level linguistic processing*..”(p. 25).

The effects of late acquisition of a first language are not limited to behavioral and cortical effects within that language. The early acquisition of a secure first language enables a second language to be learned more effectively, too. Adult hearing speakers of English can learn a sign language quickly and efficiently (Mayberry and Lock, 2003). Early and efficient language acquisition allows a language scaffold to support the learning of a subsequent language, despite the modality differences between speech and sign. The implication is that, where a language is not acquired early in life, all subsequent language skills will be at risk (Mayberry, 2007, 2010).

Capek et al. (2009) reported that deaf native signers showed a typical electrophysiological signature previously shown only for spoken language perception. This is a left anterior negativity (LAN) elicited when the perceiver encounters a syntactic anomaly within an utterance. Skotara et al. (2011) showed that deaf native sign learners when tested in their second language—written German—showed a LAN to syntactic violations: an electrophysiological pattern which characterized hearing German speakers when tested in written German. Deaf late sign learners did not show that pattern, although their written German was of similar proficiency to that of the other deaf respondents. This (sole) illustrative study of adult deaf late sign language learners points to abnormal cortical circuitry for language processing—the pattern that is typically seen in prelingually deaf CI patients who fail to benefit from CI.

Two recent studies have reported the impact of first (sign) language acquisition on speech and language outcomes following CI (Hassanzadeh, 2012; Davidson et al., 2014). These (independent) studies investigated deaf children born to deaf parents and with a native sign language background. Davidson et al. (2014) identified five young deaf children of signing parents, and compared their speech and language skills post-CI with those of 20 hearing children who used sign at home with their deaf parents (Koda), but who were also fluent English users. The groups were indistinguishable, with the CI children falling well within the range of English language skills of their hearing peers.

*“Our primary conclusion is that early knowledge of a sign language does not prevent subsequent spoken language development using a CI and that it might well lead to greater success with such development”* (Davidson et al., 2014, p. 248).

Hassanzadeh (2012) compared deaf native signers with deaf children born to hearing parents and who had no sign language experience early in life (late learners). In contrast to the predictions from an auditory-neural critical period hypothesis (Sharma et al., 2005), and in support of Davidson et al. (2014) suggestion, the deaf children who were exposed to sign language early in life had *better* speech and language outcomes following implant. While these two studies, both of which used (necessarily) small samples of deaf native signers, cannot be considered definitive, they should be considered as “straws in the wind” suggesting that successful CI outcome may be related to pre-implant sign language proficiency.

To date, sign language proficiency, assessed in terms that are comparable to those used to test the linguistic competence of a hearing user of a spoken language, have not been administered to any of the deaf patients reported in the studies of cortical organization following CI. Many of them were reported to “prefer to use sign language”. However, many deaf people, including those who acquire a sign language late, prefer to use sign rather than speech. “Uses a sign language” is no indicator of proficiency, which, to be valid, would need to interrogate phonological, morphological, lexical, syntactic and discourse skills in the language. This glaring lack in assessing sign language proficiency in prelingually deaf CI patients, 95% of whom are born to non-signing parents, means that a potential factor in the efficiency of CI for speech outcome has been ignored until now. It seems likely that an early and well-established visual language (whether sign or visible speech-based) may be critical in determining the efficiency of CI in delivering a “new” language source (auditory speech) to a language-impoverished brain. As CI is being introduced in much younger prelingually deaf patients it is unlikely that such sign language proficiency tests will be prioritized. Yet the inferences concerning the functional decoupling hypothesis, and its recommendations for shielding from non-auditory inputs, are based on studies where late language learning—and its sequelae in terms of cortical circuitry—is the norm.

## Conclusions

Sensory deprivation at birth affects two neurodevelopmental cortical processes: trophic processes which determine the disposition of critical neural structures and connections within primary sensory areas, and neuroplastic processes which reflect “compensatory” processing in regions that may be distant from primary regions (Lyness et al., 2013). Both may be time-sensitive, although with different time scales reflecting different sensitive and critical periods (Gordon et al., 2011). In prelingual deafness, we have argued that there is no good evidence that dystrophic processes within A1 are amplified by exposure to visual stimuli, including sign language, during a sensitive developmental period. As for neuroplastic processes, it is argued that, far from being essentially an auditory region, A2 is intrinsically multimodal—although in hearing people audition dominates. This being so, we find no good reason to suppose that signals from a “dormant” A1 activated by input from CI should be unable to be processed further (i.e., in association cortex including A2), as proposed by the functional decoupling hypothesis (see Lyness et al., 2013 for further discussion).

If these arguments are accepted, the psychological processes embedded in this cortical circuitry can then be elucidated. We have argued that speech itself is intrinsically multimodal, with seen as well as heard components, and that visual speech is processed by auditory cortex, including some parts of A1. Does CI impact on the *balance* of bisensory sensitivity? Possibly, but this may be a positive, not a negative, aspect of the effects of CI on the language processing network in the developing brain (Rouger et al., 2007, 2008). To date, we have very sparse data on this question.

The reported disordered cortical circuitry attributed to exposure to visual stimuli affecting auditory regions is, we suggest, more likely to be associated with disordered language learning in the sensitive early years, rather than simple exposure to visual language, be it seen speech or a sign language. Arguing from excellent outcomes for CI for postlingually deaf patients, (Giraud and Lee, 2007, p. 381) say “*First language acquisition thus appears to be a major landmark in brain organization signaling a major constraint on language-related brain plasticity”*. Similar arguments may apply when a sign language is acquired as a first language. No studies have satisfactorily demonstrated that such a language trajectory is a contraindication for CI.

Since at least 95% of children born deaf are born to hearing parents, many fail to acquire a native first language within the normal developmental time frame and to a level of proficiency comparable to that of a hearing child exposed to auditory speech. Moreover, no studies to date have examined sign language proficiency in relation to cortical organization in individuals with CI. The two studies to date which have assessed this issue by investigating outcomes for deaf children of deaf parents (i.e., children who have acquired (a sign) language within the normal developmental time frame) show good outcomes, whether contrasted with hearing children of deaf parents (Davidson et al., 2014) or deaf late language learners with CI (Hassanzadeh, 2012).

What do these arguments mean for the clinical management of CI in prelingual deafness? This is a complex issue, fraught with many difficulties including socio-political (who pays for the implant and associated support?) and bio-ethical ones (can the deaf infant be a willing patient?) in addition to those relating to language environment and education. “Native sign language acquisition” is feasible only for the very small minority of deaf children raised in multigenerational deaf communities, and there can be variability between individuals in using seen speech as a route into language. However, rather than shielding the developing infant from visual communication by seen speech and sign, we suggest that the deaf child awaiting CI may need language input of any and all sorts to enable effective language development to proceed. At least one recent behavioral study endorses this approach. Giezen (2011) investigated 15 5–6 year old deaf children (average age of CI was 1.8 years) some of whom were raised using “some form of signed communication” while others were raised in a Total Communication (TC: speech accompanied by manual signs) setting. Both groups performed equivalently on auditory speech perception and word learning tasks. Exposure to bimodal input (TC) had no deleterious effect on subsequent speech-based learning. Learning of novel signs was also tested—and showed positive correlations with new spoken-word learning: that is, exposure to bimodal input was not disadvantageous to later language learning, however this was tested.

“*These findings suggest that spoken and sign language development are not mutually exclusive for children with a CI”* (Giezen, 2011, p. 211).

The development of appropriate cortical circuitry to support the signal delivered by the implant is not, on our reading of the literature, affected by such pre-implant exposure. The early months and years are crucial for the development of language (Mayberry, 2010). With increasingly early age of implant it is more likely that the deaf infant will be able to access the speech that surrounds her. Post-implant, while auditory rehabilitation is clearly necessary to enable effective functioning of the CI, we find no compelling evidence that the rehabilitation of hearing—*on its own—*predicts satisfactory speech and language progress. Recent studies suggest that audiovisual speech rehabilitation can be as effective as auditory-only rehabilitation (Oryadi Zanjani et al., 2013), while retrospective studies on early- and late-implanted patients suggest that post-implant language success is related to the extent of exposure to speech in the home (Boons et al., 2012).

Early CI is a remarkable breakthrough in delivering hearing to the child born deaf, but its success needs to be evaluated beyond its auditory impact. Where neurophysiological and neuroimaging evidence has been brought to bear on CI outcome success, inferences have often been in relation to the impact of congenital deafness on auditory brain circuitry, ignoring the overall picture of language development in the deaf child. Our view of the evidence suggests that good first language acquisition within the early years, however that may be achieved, may be the best predictor of successful language outcome for the child born deaf.

## Conflict of interest statement

The authors declare that the research was conducted in the absence of any commercial or financial relationships that could be construed as a potential conflict of interest.

## Footnotes

^1^This became clear before the era of pediatric CI. The Tadoma method, which “remaps” previously heard sound frequencies as felt vibrations of the talker’s vocal tract, can restore useful speech perception in children who become deaf after the age of 18 months (Chomsky, 1986).

^2^Functional decoupling in the auditory system refers to the process of re-organization of integrative regions of auditory cortex, when congenital deafness has led to failure of activation within primary auditory areas. Functionally, the effect is thought to be a disconnection between the processing of the auditory signal in primary projection sites and further processing of the signal which cannot be remedied if CI is performed after the critical developmental period has elapsed (see Lyness et al., 2013 for further discussion).

^3^see http://www.cochlear.com

^4^None of this sample used a signed language and their speechreading skills were “..*insufficient to permit communication (outside) the family*..” (Lee et al., 2007a).

^5^In postlingually deaf children, hypometabolism in A1 and A2 can be associated with better CI outcome—see Lazard et al. (2011).

^6^It is possible to criticize those studies that failed to find visual activation in A1 on the basis of insufficient power in the analysis, or misleading confirmation of the null hypothesis (type-2 error). However, Leonard et al. (2012) used MEG constrained by individual fMRI to isolate both the time-course and cortical localization for stages of language processing to explore this issue directly. They examined sign language processing in deaf native signers and contrasted this with speech processing in hearing non-signers. Lexico-semantic processing activated a left superior temporal network equivalently in deaf signers and hearing speakers. Signs did not activate primary auditory cortex in deaf signers. In people with normal hearing A1 was activated by speech.

^7^There is also plentiful evidence, both from these and from earlier studies (Giraud et al., 2001; Giraud and Truy, 2002; Kang et al., 2004; Doucet et al., 2006; Lazard et al., 2011) that cortical visual areas are recruited to a greater extent in CI implantees than in people with normal hearing. These studies, while demonstrating the relative salience and long-term impact of visual processing in deaf people (including CI implantees) do not affect the argument here, but they do suggest that prior to implantation, deaf people develop a range of skills that reflect recruitment of visual networks to the task of speech processing.

^8^A more appropriate contrast might be with people who are hard-of-hearing, given that the CI signal is degraded with respect to the spectral components of speech. Such comparisons are lacking at present (but see McGettigan et al., 2012; for explorations of cortical correlates of degraded audition and vision in speech processing in people with normal hearing). Claims that CI patients, even those who are successful in using the CI to identify auditory speech, never develop “normal” audiovisual processing skills (e.g., Suh et al., 2009; Tremblay et al., 2010) may need to be moderated when this contrast is taken into account.

^9^Here we include systems such as Visual Phonics or Cued Speech (LaSasso and Crain, 2010) where manual actions alongside seen speech provide phonological information. These can be used in communication between hearing caregivers and deaf infants from an early age. If cued speech is acquired as a first language, skilled speech perception can approach that of a child with normal hearing (Leybaert and LaSasso, 2010). Structural neuroanatomical studies, focussing on gray matter volumes and connectivity within opercular systems, suggest that brain structure in such early deaf speechreaders closely resembled that of hearing controls (Olulade et al., 2014).
